# Understanding voice disorders among university staff: prevalence and associated factors

**DOI:** 10.1186/s12889-026-27088-8

**Published:** 2026-04-13

**Authors:** Faten Ezzelarab Younis, Sally Abdelwanees, Eman Fathi El-Domiaty, Amira Mohamed Abdel-Monaem, Norhan Moussa, Shaimaa Yaihya Abdel-Raouf

**Affiliations:** 1 Public Health and Community Medicine Department, Faculty of Medicine, Menoufia University, Menoufia governorate, Shebin El-Kom city, Egypt; 2 Phoniatrics Unit, Otorhinolaryngology Department, Faculty of Medicine, Menoufia University, Menoufia governorate, Shebin El-Kom city, Egypt; 3 Private International Law Department, Faculty of Law- Menoufia University, Menoufia governorate, Shebin El- Kom city, Egypt

**Keywords:** Voice disorders, University staff, Associated factors, VHI-10, Videolaryngostroboscopy

## Abstract

**Background:**

Voice disorders are a common health problem, especially among university staff members due to their excessive voice use. It might have significant consequences on their quality of life and work productivity. So, this study aimed to determine the prevalence of voice disorders among Egyptian staff members at Menoufia University and identify the contributing factors.

**Methods:**

This cross-sectional study included 400 university staff members from different faculties in Egypt’s Menoufia University, recruited through convenience sampling. The participants obtained a self-administered questionnaire including sociodemographic information, occupational details of teaching, voice complaints, and the Voice Handicap Index-10 (VHI-10). A videolaryngostroboscopic examination was performed to identify the pathology.

**Results:**

The main voice complaints among the university staff were dysphonia (40.3%) and throat dryness (41%). An abnormal VHI-10 was obtained in 164/400 (41.0%) of participants. The videolaryngostroboscopy revealed functional voice disorders (43.1%), minimal associated pathological lesions (30.4%), organic voice disorders (20.6%), and combined ones (5.9%). The significant associated factors for abnormal VHI were frequent teaching sessions per week (aOR: 1.25, 95% CI: 1.14–1.36), working a job ranked as teaching staff (aOR: 2.67, 95% CI: 1.19–5.96), deficient water intake of less than three cups per day (aOR: 5.09, 95% CI: 2.39–10.87), lack of use of amplifiers (aOR: 1.92, 95% CI: 1.15–3.19), and positive family history (aOR: 2.54, 95% CI: 1.09–5.91).

**Conclusions:**

Voice disorders are highly prevalent among university staff members. Voice disorders were significantly associated with several personal, occupational, and behavioral factors. These emphasize the significance of implementing a health education program about voice care and putting practical preventive strategies into practice.

**Supplementary Information:**

The online version contains supplementary material available at 10.1186/s12889-026-27088-8.

## Introduction

Voice disorders (VDs) refer to abnormalities in voice production or lack of quality, tone, volume, resonance, or continuity that are improper for the person’s age and gender [1]. Voice disorders are a common occupational health problem among professional voice users, like teachers and university staff, as their voice is the principal tool for their work, and they usually use it at a high volume [2]. These VDs can significantly impact both their quality of life and work performance, ultimately leading to a decrease in work productivity [2–4]. A recent systematic review and meta-analysis documented that the occupational voice complaint frequency among voice users was 43.9% [5]. Approximately 92% of Egyptian faculty members lacked adequate knowledge concerning voice care [6].

There are several kinds of risk factors for VDs, including sociodemographic traits like smoking, coffee drinking, and female gender; occupational factors like loud voice use, long teaching hours, and noisy environments; or medical conditions like trauma, allergy, or surgery [7–10].

There are several self-reported questionnaires to assess voice disorders, including the Voice Handicap Index (VHI) [11], the Voice-Related Quality of Life (V-RQOL) [12], the Voice Symptom Scale (VoiSS) [13], and Vocal Tract Discomfort Scale (VTDS) [14]. The Voice Handicap Index was a thirty-item tool that was created by Jacobson in 1997 to quantify the degree to which a voice problem affects three aspects of a patient’s life: functional, physical, and emotional levels [11]. In 2004, Rosen et al. created the Voice Handicap Index-10 (VHI-10) to be a more condensed form that patients can complete in a short time without sacrificing validity [15]. Arffa et al. in 2012 reported the normative scores for the VHI-10 tool [16]. The Arabic versions of V-RQOL [17] and the Vocal Tract Discomfort Scale (VTDS) [18] were used by previous studies [2, 9, 19–21]. The Voice Handicap Index-10 (VHI-10) is a validated, widely used, and reliable screening tool. In addition, it is brief and easy to administer, which makes it suitable for large population-based studies [15]. In 2012, Farahat validated an Arabic version of the VHI-10 and found excellent test-retest reliability (*r* = 0.920) and great internal consistency with Cronbach’s α of 0.88 [22].

Videolaryngoscopy is a useful diagnostic instrument that assists in the management of VDs by evaluating the pathological findings among those who have them [23]. It aids visualization of the larynx and vocal folds’ structure and function. It is a distinct clinical examination to verify and assess VDs [24].

In Egypt, research on VDs among university staff, as opposed to teachers, is scarce and only evaluates subjective instruments without identifying the underlying causes using diagnostic techniques to assist the professional teaching staff in resolving their disorders. Several previous studies conducted in Egypt and other Arabic-speaking populations have relied primarily on subjective voice assessment tools [2, 9, 19–21]. Therefore, the purpose of this study was to determine the prevalence of voice disorders among university staff at Menoufia University in Egypt using VHI-10 and videolaryngostroboscopic examination and to identify associated factors including sociodemographic, occupational, behavioral, and genetic characteristics.

## Materials and methods

### Study design, timing, and setting

This cross-sectional study was conducted from the beginning of July to the end of November 2025 at the Menoufia University Faculties in Egypt.

### Calculation of sample size

Using the OpenEpi online calculator, version 3, the sample size was calculated at 80% power with a 0.05 α error. Based on a previous study in Saudi Arabia [7], which found that 38.8% of academic staff overall had an abnormal VHI, an estimated 365 individuals were included. 15% was added for the non-response rate; there were 420 in total.

*Participants*: Menoufia University in Egypt has twenty colleges, with a 1:3 ratio of five medical and fifteen non-medicals. Simple random sampling was performed using a computer-generated randomization method to select one medical faculty and three non-medical faculties. The questionnaire was distributed to university staff members, and responses were collected until the determined sample size was reached using a convenience sampling method. The total university staff who participated was 400, with a 95.2% response rate.

### Inclusion and exclusion criteria

Each staff member, regardless of age or gender, was engaged. A history of neck trauma or injury, a thyroidectomy or other neck surgery that impacted a person’s voice, or a general anesthesia procedure that affected the voice or neck tumor were also ineligible criteria.

### Tools

- The questionnaire was divided into two sections, and its completion took approximately 10 min per participant through personal interviews:
- The first part included items about sociodemographic and occupational data, habits that may affect voice, teaching-related factors, medical history, and awareness about voice care (as illustrated in the supplementary file). These questions were pilot tested on 37 university staff members at Menoufia University in Egypt to validate this section. Feedback from the pilot study was used to revise items for clarity and comprehensibility. A panel of experts in phoniatrics and epidemiology guaranteed content validity. Participants involved in the pilot testing were excluded from the final study sample.
- The second part comprised a previously validated instrument: the Arabic version of the Voice Handicap Index-10 (VHI-10), adopted from a prior study [22]. It consisted of ten questions, each of which had a score between 0 and 4 (0 denoting never, 1 nearly never, 2 sometimes, 3 almost always, and 4 always). The VHI-10 had a maximum score of 40; a score of more than 11 was considered abnormal [16].
- All the participants who had abnormal VHI-10 (164 participants) were invited to laryngeal examination by videostroboscopy at Phoniatrics Clinic of Menoufia University Hospital; only 102 of them responded (62.2%). Videostroboscopy was performed using a 70-degree angle rigid laryngoscope (Shenda, China), Invisia’s MediCam Plus camera with a high-sensitivity charge-coupled device sensor (1280 × 960 pixels), and a fiber-optic light cable (4.8 × 1800 mm). The Inventis Daisy software system 3.6.3B3, which includes the Visia module created by the Inventis Company, is utilized. In both normal and stroboscopic light, vocal fold function was evaluated while the subject was at rest and while pronouncing the sustained/i/vowel at a comfortable pitch and loudness.

Diagnosed laryngeal pathologies were classified into three main categories according to the occurrence of noticeable morphological changes [25]: (1) Organic voice disorders, which result from changes in the mechanism of voice production, including respiratory, laryngeal, or vocal tract (e.g., inflammatory, neurological, and traumatic disorders). (2) Functional (non-organic) voice disorders in which the physical character is intact while faulty use of voice leads to these disorders, e.g., hyperfunction dysphonia, hypofunction dysphonia, phonasthenia, and ventricular dysphonia. (3) Minimal Associated Pathological Lesions (MAPLs), which include small benign lesions affecting vocal folds, e.g., vocal fold nodules, polyps, Reinke’s edema, polypoid degeneration, laryngeal cysts, and contact granulomas.

### Statistical analysis

The statistical analyses were performed using SPSS software (version 24.0, Chicago, IL, USA). The relationships between dichotomous variables were examined using the chi-square test (χ²). When comparing two quantitative data sets, the student’s t-test or Mann-Whitney was used as needed. To find independent factors for abnormal VHI, binary logistic regression was employed. A P-value of less than 0.05 was set as the significance level.

## Results

Four hundred staff members from Menoufia University’s various faculties participated in this cross-sectional study. Their mean age was 41.42 ± 7.76, with a range of 24–81 years old; 86.8% of them were females, and 77.5% of them were urban residents. Of them, 87.3% were married, and 85.3% had children. Teaching staff made up the majority of the degree rank (86.3%), with assistant staff making up just 13.8%. Amongst the medical staff, 66.7% were teaching basic science subjects. Of them, 63.0% used speakers, and 52.0% had participated in remote learning. The number of teaching sessions each week ranged from one to twenty, and the employment years ranged from one to fifty years (Table 1).

**Table 1:** Relationship between voice handicap index (VHI) and other parameters

Parameter	Total participants (*N* = 400)	Abnormal VHI (*N* = 164)	Normal VHI (*N* = 236)	*P* value
Gender				
Male	53 (13.3%)	13 (7.9%)	40 (16.9%)	0.01*
Female	347 (86.8%)	151 (92.1%)	196 (83.1%)	
Age (years)				
Mean ± SD	41.42 ± 7.76	41.01 ± 6.72	41.69 ± 8.41	0.37
Range	24–81	27–62	24–81	
Residence				
Urban	310 (77.5%)	123 (75.0%)	187 (79.2%)	0.31
Rural	90 (22.5%)	41 (25.0%)	49 (20.8%)	
Marital status				
Married	349 (87.3%)	151 (92.1%)	198 (83.9%)	0.02*
Unmarried	51 (12.8%)	13 (7.9%)	38 (16.1%)	
Have children				
Yes	341 (85.3%)	148 (90.2%)	193 (81.8%)	0.02*
No	59 (14.8%)	16 (9.8%)	43 (18.2%)	
Number of children				
0	59 (14.8%)	16 (9.8%)	43 (18.2%)	
1–3 children	289 (72.3%)	123 (75.0%)	166 (70.3%)	0.04*
≥4 children	52 (13.0%)	25 (15.2%)	27 (11.4%)	
Occupation				
Assistant staff	55 (13.8%)	13 (7.9%)	42 (17.8%)	0.01*
Teaching staff	345 (86.3%)	151 (92.1%)	194 (82.2%)	
Faculty				
Medical	234 (58.5%)	103 (62.8%)	131 (55.5%)	0.14
Non-medical	166 (41.5%)	61 (37.2%)	105 (44.5%)	
Medical staff	*N* = 234	(*N* = 103)	(*N* = 131)	
Basic	*156 (66.7%)*	75 (72.8%)	81 (61.8%)	0.07
Clinical	*78 (33.3%)*	28 (27.2%)	50 (38.2%)	
Use of amplifiers				
Yes	252 (63.0%)	87 (53.0%)	165 (69.9%)	0.001*
No	148 (37.0%)	77 (47.0%)	71 (30.1%)	
Remote learning				
Yes	208 (52.0%)	92 (56.1%)	116 (49.2%)	0.17
No	192 (48.0%)	72 (43.9%)	120 (50.8%)	
Teaching session/week				
Median (range)	2.5 (1–20)	4 (1–20)	2 (1–20)	< 0.001*
Years of employment				
Median (range)	14 (1–50)	15 (2–35)	14 (1–50)	0.26
Smoking				
Yes	23 (5.8%)	11 (6.7%)	12 (5.1%)	0.47
No	377 (94.3%)	153 (93.3%)	224 (94.9%)	
Use loud voice				
Yes	258 (64.5%)	116 (70.7%)	142 (60.2%)	0.03*
No	142 (35.5%)	48 (29.3%)	94 (39.8%)	
Excess tea (> 3cups/day)				
Yes	55 (13.8%)	24 (14.6%)	31 (13.1%)	0.66
No	345 (86.3%)	140 (85.4%)	205 (86.9%)	
Excess coffee (> 3cups/day)				
Yes	38 (9.5%)	10 (6.1%)	28 (11.9%)	0.05
No	362 (90.5%)	154 (93.9%)	208 (88.1%)	
Water/day				
<3 cups	82 (20.5%)	37 (22.6%)	45 (19.1%)	< 0.001*
4–6 cups	211 (52.8%)	110 (67.1%)	101 (42.8%)	
>6 cups	107 (26.8%)	17 (10.4%)	90 (38.1%)	
GERD				
Yes	220 (55.0%)	101 (61.6%)	119 (50.4%)	0.02*
No	180 (45.0%)	63 (38.4%)	117 (49.6%)	
Allergic diseases, especially respiratory				
Yes	73 (18.3%)	28 (17.1%)	45 (19.1%)	0.69
No	327 (81.8%)	136 (82.9%)	191 (80.9%)	
Chronic sinusitis or rhinitis				
Yes	192 (48.0%)	90 (54.9%)	102 (43.2%)	0.02*
No	208 (52.0%)	74 (45.1%)	134 (56.8%)	
Chronic cough				
Yes	17 (4.3%)	4 (2.4%)	13 (5.5%)	0.13
No	383 (95.8%)	160 (97.6%)	223 (94.5%)	
Repeated respiratory tract infection				
Yes	45 (11.3%)	15 (9.1%)	30 (12.7%)	0.26
No	355 (88.8%)	149 (90.9%)	206 (87.3%)	
Family history for voice disorders				
Yes	36 (9.0%)	25 (15.2%)	11 (4.7%)	< 0.001*
No	364 (91.0%)	139 (84.8%)	225 (95.3%)	

*Significant difference; *SD*, standard deviation; *GERD*, gastroesophageal reflux disease

Abnormal VHI was present among 164 out of 400 university staff (41.0%). When sociodemographic characteristics were compared among those with abnormal VHI and those with normal VHI, female gender, being married, and having children were associated with a higher frequency of abnormal VHI (*P* < 0.05). Regarding occupational history, the prevalence of abnormal VHI was higher among teaching staff than assistant staff (*P* < 0.05), but there was no significant difference in work years or occupational history between medical and non-medical staff members (*P* > 0.05). Additionally, abnormal VHI was more common for those who attended sessions more frequently each week and did not use amplifiers (*P* < 0.05) (Table 1).

Regarding habits, it was found that those who used a loud voice and drank deficient water had a higher frequency of abnormal VHI than others (*P* < 0.05). Asking about medical history, it was found that the presence of gastroesophageal reflux disease (GERD) and chronic sinusitis or rhinitis among studied staff members was significantly associated with abnormal VHI (*P* < 0.05). Also, those who had a positive family history of voice disorders showed a significantly high prevalence of abnormal VHI (*P* < 0.05) (Table 1).

**Fig. 1:**
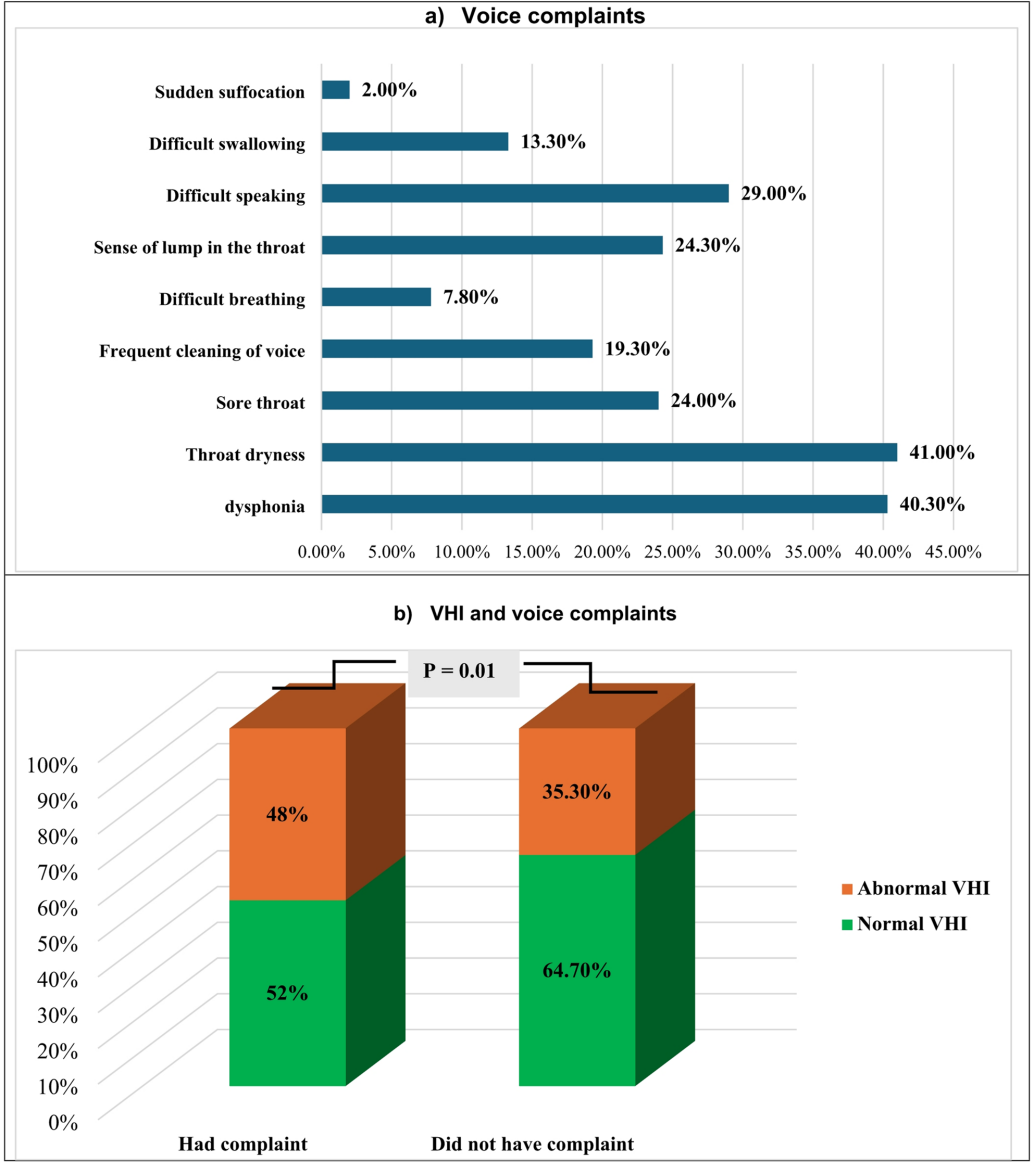
The voice complaints and its voice handicap index (VHI) among the studied university staff members

Throat dryness and dysphonia were the most common voice complaints among the participants under study (41.0% and 40.3%), followed by difficulty speaking, sense of lump in the throat, sore throat, frequent cleaning of voice, and difficult swallowing (29%, 24.3%, 24%, 19.3%, and 13.3%, respectively). However, difficult breathing and sudden suffocation were the less frequent voice complaints (7.8% and 2%, respectively) (Fig. 1a). The prevalence of abnormal VHI was greater in those who had complained than in those who had not (48% versus 35.3%, *P* < 0.05) (Fig. 1b).

Among the 179 participants (44.8%) who reported having voice complaints, the consequences varied from no effects (63.7%) to absence from work (33%) and thinking about leaving work (3.3%). The participants used various methods to alleviate these complaints, the most common ones being excess intake of fluids (95.0%), avoiding shouting (95.0%), and avoiding noisy environments (88.8%). Only 31.8% of them went to a doctor for these complaints. Those who did not seek medical advice had many reasons: 33.6% of them stated they didn’t have enough time, 100% said the condition was not a serious condition, and 4.1% mentioned they did not know how and where to seek medical advice. Regarding awareness about voice care, only 28.5% of the studied group were aware of this, and their sources of information were nearly equally distributed between the internet, family & friends, doctors’ visits, and books, but training courses were a smaller number (7.0%). When asked about their intention to attend training courses about voice care whenever available, most of them (77.0%) said they would attend (Table 2).

**Table 2:** Voice complaints and awareness about voice care

Parameter	Total participants (*N* = 400)
Its consequences on professional life	*N* = 179
• No effect	*114 (63.7%)*
• I had thought to leave work	*6 (3.3%)*
• Take absenteeism from work	*59 (33.0%)*
Presence of voice complaints	
Yes	179 (44.8%)
No	221 (55.3%)
Did you do any of these methods to relieve your complaint?	*N* = 179
• Excess intake of fluids	*170 (95.0%)*
• Avoid shouting	*170 (95.0%)*
• Avoid noisy environment	*159 (88.8%)*
• Use speakers	*109 (60.9%)*
• Visit a doctor	*57 (31.8%)*
If you didn’t visit a doctor, what was the cause?	*N* = 122
• I do not have enough time	*41 (33.6%)*
• It is not serious	*122 (100%)*
• I don’t know how or where to seek medical help	*5 (4.1%)*
Did you hear about voice care?	
Yes	114 (28.5%)
No	286 (71.5%)
Source of your information about voice care	
Internet	85 (21.3%)
Family & friends	68 (17.0%)
Doctors visited for voice disorders	55 (13.8%)
Social media	67 (16.8%)
Training courses	28 (7.0%)
Books	68 (17.0%)
If available, are you ready to attend courses about voice care	
Yes	308 (77.0%)
No	92 (23.0%)

By regression analysis, the significant predictors for participants’ abnormal VHI were frequent weekly teaching sessions (aOR: 1.25 with 95% CI: 1.14–1.36, *P* < 0.05), being a member of the teaching staff (aOR: 2.67 with 95% CI: 1.19–5.96, *P* < 0.05), consuming less than three to six cups of water daily (for 3 cups/day, aOR: 5.09 with 95% CI: 2.39–10.87 and for 4–6 cups/day, aOR: 4.10 with 95% CI: 2.14–7.86, *P* < 0.05), not using amplifiers (aOR: 1.92 with 95% CI: 1.15–3.19, *P* < 0.05), and having a positive family history of voice disorders (aOR: 2.54 with 95% CI: 1.09–5.91, *P* < 0.05) (Table 3).

**Table 3:** Binary logistic regression to detect predictors for voice disorders

Parameter	β	aOR	95% CI	*P* value
Marital status				
Married				
Unmarried (reference)	0.91	2.49	0.44–14.01	0.30
Gender				
Male (reference)	0.17	1.19	0.53–2.63	0.67
Female				
Having children				
Yes	0.92	2.50	0.50–12.41	0.26
No (reference)				
Teaching sessions per week	0.22	1.25	1.14–1.36	< 0.001*
Occupation				
Assistant staff (reference)	0.98	2.67	1.19–5.96	0.02*
Teaching staff				
Use of loud voice				
Yes	0.33	1.39	0.83–2.32	0.21
No (reference)				
Water/day				
< 3 cups	1.62	5.09	2.39–10.87	< 0.001*
4–6 cups	1.41	4.10	2.14–7.86	< 0.001*
> 6 cups (reference)				
Use of speakers				
Yes (reference)	0.65	1.92	1.15–3.19	0.01*
No				
Family history for voice disorders				
Yes	0.93	2.54	1.09–5.91	0.03*
No (reference)				
Chronic sinusitis or rhinitis				
Yes	0.12	1.13	0.68–1.87	0.64
No (reference)				
GERD				
Yes	0.28	1.32	0.81–2.17	0.27
No (reference)				

*Significant difference; *β,* Beta; *aOR,* adjusted odds ratio; *CI,* Confidence Interval

**Fig. 2:**
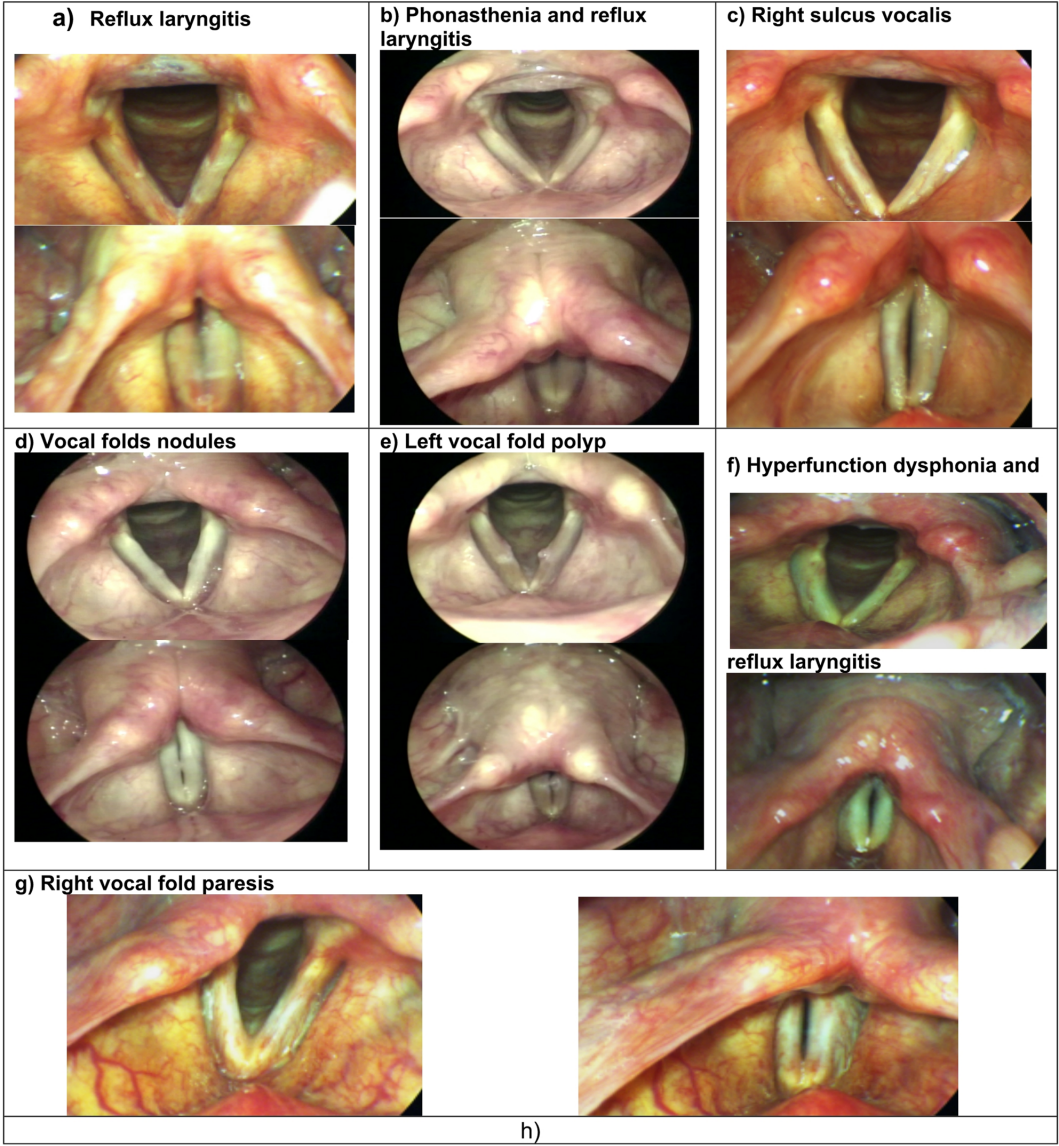
The pathological findings of videolaryngostroboscopy and the voice disorders among the participants who had abnormal voice handicap index (VHI) h) The voice disorders by videolaryngostroboscopy

Videolaryngostroboscopic examination among those who had abnormal VHI revealed various pathologies such as reflux laryngitis, vocal fold polyp, phonasthenia, right sulcus vocalis, vocal fold nodules, hyperfunction dysphonia, and vocal fold paresis (Fig. 2). Based on these pathological findings, the voice disorders among those who had abnormal VHI were classified as follows: functional voice disorders (43.1%), MAPLs (30.4%), organic voice disorders (20.6%), and combined ones (5.9%) (Fig. 2). Videolaryngostroboscopy revealed normal laryngeal findings in 8.8% of participants who had abnormal VHI-10 scores and were diagnosed with phonasthenia.

## Discussion

This cross-sectional study aimed to evaluate the prevalence of voice disorders among university staff across different faculties at Menoufia University using the VHI-10 questionnaire and videolaryngostroboscopic examination, and to investigate the associated factors. A sample of four hundred Menoufia University staff participated in this cross-sectional study. Approximately 40% of all staff members under study exhibited abnormal VHI-10. It agrees with a prior study conducted on academic staff workers in Saudi Arabia, which discovered that 38.8% of participants had abnormal VHI-10 [7].

Among the studied participants, 179 (44.8%) had voice complaints. It is similar to a comprehensive study and meta-analysis; 43.9% of voice users reported having vocal complaints [5].

The main complaints among the studied group were throat dryness and dysphonia. The participants who suffered from voice complaints had greater abnormal VHI than those who did not suffer. Similarly, a previous study conducted in Iran showed that the main complaints were vocal fatigue and hoarseness, with a significant difference between the complaining and non-complaining groups regarding the VHI scale [26]. This association between voice complaints and high VHI scores (abnormal VHI) could be explained by the fact that vocal complaints can interfere with daily or social activities, which raises the VHI score.

More than 60% of the participants with abnormal VHI responded to examination by videolaryngostroboscopy. A variety of pathological changes, including reflux laryngitis, vocal fold polyps, nodules or paresis, phonasthenia, sulcus vocalis, and hyperfunction dysphonia, were found during this examination. These alterations were used to classify VDs into four categories: organic VDs, functional VDs, MAPLs, and mixed lesions. These results align with previous research on professional voice users [27–29]. Functional VDs were the most frequent laryngeal pathology; it was in line with a previous study conducted on an academic population [30]. Another study by Preciado-López et al. reported that the prevalence of voice disorders among teaching staff was 57.1%: 28.8% for functional dysphonia, 20.25% for organic dysphonia, and 8.1% for chronic laryngitis [31].

Of the participants in the study, less than one-third knew information about voice care. This was consistent with earlier studies [6, 32]. It demonstrates how important it is to implement a health education program that helps these participants understand the healthy habits necessary to maintain their voice.

This investigation reported that different sociodemographic characteristics were significantly associated with abnormal VHI, such as female gender; this was in line with prior studies [31, 32]. It may be attributed to the vocal fold containing hormonal receptors, and female hormones can impact the laryngeal neuromuscular functions [33]. On the other hand, previous studies’ results showed no relationship between voice abnormalities and gender [7, 10]. Therefore, additional research concerning gender variation with voice disorders is required.

Also, those who married, had children, and worked a job ranked as teaching staff were significantly associated with abnormal VHI, which may be attributed to excessive voice utilization. In contrast, a previous study in Saudi Arabia [2] reported no association with marital status, having children, or the academic job rank.

Nevertheless, there was no significant association found in this study between smoking and abnormal VHI, like the findings of previous research [2, 10]. On the contrary, a prior study indicated that among Spanish teaching professionals, smoking was a significant risk associated with voice abnormalities [31]. Most of the participants in this study were female staff who did not smoke, which might be to blame for this diversity.

Among habitual and medical characteristics of the studied participants, the use of a loud voice, inadequate water intake, GERD, and chronic sinusitis were significantly associated with abnormal VHI. It agrees with previous studies that found the significant association between abnormal VHI with loud voice and medical history [2, 7, 9].

After adjusting for all independent variables in binary logistic regression, all of these sociodemographic characteristics were not significant predictors of abnormal VHI; however, occupational, behavioral, and genetic characteristics, such as job rank as a teaching staff member, the frequency of weekly teaching sessions, the absence of amplifier use, insufficient water consumption, and positive family history, were significant predictors. This is in keeping with a previous study conducted in Saudi Arabia, which determined that all sociodemographic characteristics were not significant factors for the perception of voice abnormality [2].

Frequent intake of water was found to be associated with normal VHI; this is supported by a systematic review done by Alves et al. [34], which had found that increased water intake led to significant improvement in phonation. Also, use of speakers as microphones was a protective factor for voice abnormality. It is supported by a previous trial that approved the role of amplification system use as a preventive measure for VHI abnormality [35].

There was a strong association between voice abnormality and both the number of weekly teaching sessions and a positive family history. It was in line with a prior study [32]. It could be attributed to genetics that could result in cellular damage to the vocal folds, which would be exacerbated by occupations that demand extensive voice use [36].

## Conclusion

The prevalence of voice problems among the staff members under study was high, and few of them were aware of voice care. The most important contributing factors were frequent weekly teaching sessions, position as a teaching staff member, inadequate water intake, lack of speaker use, and a positive family history. These highlight the necessity of implementing a health education program for voice care to lessen this problem. Additionally, conduct routine VHI and videolaryngostroboscopy examinations for university staff who exhibit anomalies.

## Strengths and limitations

The VHI-10, a validated instrument, was used in this study to identify voice disorders among university staff members; therefore, the results are more accurate than self-reporting the severity of voice problems. As well as the videolaryngostroboscopy examination, it was utilized to diagnose the underlying pathology and categories of VDs. It listed several associated factors. There were certain restrictions. As a cross-sectional study, it only shows associations rather than causative links. Moreover, the use of convenience sampling limits the generalizability of the findings. It was suggested that more long-term studies, such as cohorts or experiments, be conducted to detect the causal relationship and assess the implications of different preventive measures.

## Supplementary Information

Supplementary Material 1

## Data Availability

Data will be available upon a reasonable request.

## Abbreviations

VDs: Voice Disorders
VHI-10: Voice Handicap Index-10
aOR: adjusted Odds Ratio
CI: Confidence Interval
MAPLs: Minimal Associated Pathological Lesions
GERD: Gastroesophageal Reflux Disease

## References

[1] American Speech-Language-Hearing Association. Definitions of Communication Disorders and Variations. Last accessed on 10 December 2025. Available online: https://www.asha.org/policy/RP1993-00208/.

[2] Al Awaji NN, Alghamdi KA, Alfaris AM, Alzamil RZ, Alhijji LN, Alyehya GS, et al. Measuring perceived voice disorders and quality of life among female university teaching faculty. J Pers Med. 2023;13(11):1568. 10.3390/jpm13111568. 10.3390/jpm13111568 PMC10672704 38003883

[3] Fageeh YA, Alotaibi TA, Althobaiti NSA, Alkhaldi AA, Althobaiti AA, Althobaiti HA, et al. Voice disorders among teachers in Taif City, Kingdom of Saudi Arabia. Cureus. 2024;16(2):e54561. 10.7759/cureus.54561. 10.7759/cureus.54561 PMC10957102 38516420

[4] Gomes NR, Teixeira LC, de Meiros AM. Vocal symptoms in university professors: their association with vocal re-sources and with work environment. J Voice. 2020;34:352–7. 10.1016/j.jvoice.2018.10.010. 10.1016/j.jvoice.2018.10.010 30473269

[5] Oliveira P, Ribeiro VV, Constantini AC, Cavalcante MEOB, Sousa MDS, da Silva K. Prevalence of work-related voice disorders in voice professionals: systematic review and meta-analysis. J Voice. 2025;39(1):84–104. 10.1016/j.jvoice.2022.07.030. 10.1016/j.jvoice.2022.07.030 36057482

[6] Mahmoud NF, Khaled DMF, Mohammed HO. Knowledge of Egyptian faculty members about voice care: a national cross‑sectional study. Egypt J Otolaryngol. 2022;38:1–9. 10.1186/s43163-022-00247-5.

[7] Ahmed EE, Bukhari MA, Melibary RA. Voice disorders among academic staff at king saud university medical college (comparison between basic science and clinical staff). J Otolaryngol ENT Res. 2017;8(1):394–6. 10.15406/joentr.2017.08.00233.

[8] Azari S, Aghaz A, Maarefvand M, Ghelichi L, Pashazadeh F, Shavaki YA. The prevalence of voice disorders and the related factors in university professors: a systematic review and meta-analysis. J Voice. 2024;38(5):1103–14. 10.1016/j.jvoice.2022.02.017. 10.1016/j.jvoice.2022.02.017 35422355

[9] Alharbi NS, Alotaibi S, Alnughaythir AI, Abohelaibah F, Alruways AQ, Alharbi R, et al. Prevalence and risk factors of voice disorders among teachers in Saudi Arabia. Cureus. 2024;16(3):e56540. 10.7759/cureus.56540. 10.7759/cureus.56540 PMC11026995 38646382

[10] Ghayoumi-Anaraki Z, Heidarian-Miri H, Zainaee S, Rahmani S, Haresabadi F, Effati M, et al. Prevalence and risk factors of voice disorders in university teaching faculty members: a pilot study. JRSR. 2020;7(4):173–7.

[11] Jacobson B, Johnson A, Grywalski C, et al. Voice Handicap Index (VHI): development and validation. Am J Speech Lang Pathol. 1997;6:66‒70.

[12] Hogikyan ND, Sethuraman G. Validation of an instrument to measure voice-related quality of life (V-RQOL). J Voice. 1999;13(4):557–69. 10.1016/s0892-1997(99)80010-1 10622521

[13] Deary IJ, Wilson JA, Carding PN, MacKenzie K. VoiSS: a patient-derived voice symptom scale. J Psychosom Res. 2003;54(5):483–9. 10.1016/s0022-3999(02)00469-5 12726906

[14] Lopes LW, de Oliveira Florencio V, Silva POC, da Nóbrega E Ugulino AC, Almeida AA. Vocal tract discomfort scale (VTDS) and voice symptom scale (VoiSS) in the evaluation of patients with voice disorders. J Voice. 2019;33(3):381.e23-381.e32. 10.1016/j.jvoice.2017.11.018. 10.1016/j.jvoice.2017.11.018 29306526

[15] Rosen CA, Lee AS, Osborne J, Zullo T, Murry T. Development and validation of the voice handicap index-10. Laryngoscope. 2004;114(9):1549–56. 10.1097/00005537-200409000-00009. 10.1097/00005537-200409000-00009 15475780

[16] Arffa RE, Krishna P, Gartner-Schmidt J, Rosen CA. Normative values for the voice handicap index-10. J Voice. 2012;26(4):462–5. 10.1016/j.jvoice.2011.04.006. 10.1016/j.jvoice.2011.04.006 21816570

[17] Abdelhay SA, Abdelgoad A, Elawady MA, Khaled DMF. The validation of the voice-related quality of life (V-RQOL): Arabic version. Egypt J Ear Nose Throat Allied Sci. 2022;23(23):1–6.

[18] Darawsheh WB, Shdaifat A, Natour YS. Validation of the Arabic version of vocal tract discomfort scale. Logoped Phoniatr Vocol. 2020;45(2):82–90. 10.1080/14015439.2019.1630481. 10.1080/14015439.2019.1630481 31244363

[19] EL‑Ghoneimy SM, Khalifa MA. Prevalence of voice disorders and associated factors among primary school teachers in Alexandria, Egypt. Alexandria J Med. 2017;53(4):363–70.

[20] Abdel-Hamid MA, Fahmy VF, Momen MA, Elokda EE. Prevalence, risk factors and impact of voice disorders among primary school teachers in Cairo, Egypt. Egypt J Community Med. 2020;38(1):33–41. 10.21608/ejcm.2020.68618.

[21] Alanazi R, Alrahim A, Bayounos S, Al-Ghuwainem A, Al-Bar MH. Association between voice handicap index and reflux symptom index: a cross-sectional study of undiagnosed general and teacher cohorts in Saudi Arabia. Sultan Qaboos Univ Med J. 2018;18(3):e350–4. 10.18295/squmj.2018.18.03.014 PMC6307638 30607277

[22] Farahat M. Validation and reliability of Arabic voice handicap index-10. Saudi Journal of Otorhinolaryngology Head and Neck Surgery. 2012;14(1):11–8. 10.4103/1319-8491.274765.

[23] Vindrani N, Patel M, Gaur S, Singh V. Role of videolaryngoscopy in patients with hoarseness of voice. Santosh University Journal of Health Sciences. 2019;5(2):109–12. 10.18231/j.sujhs.2019.023.

[24] Sulica L. Laryngoscopy, stroboscopy and other tools for the evaluation of voice disorders. Otolaryngol Clin North Am. 2013;46(1):21–30. 10.1016/j.otc.2012.09.001. 10.1016/j.otc.2012.09.001 23177402

[25] Kotby MN. The accent method of voice therapy. San Deigo: Singular publishing group; 1995.

[26] Azari S, Amiri Shavaki Y, Ghelichi L, Moossavi A, Saneii SH. Voice handicap index in Iranian rehabilitation professors with and without vocal complaints. Middle East Journal of Rehabilitation and Health Studies. 2022;9(3):e121048. 10.5812/mejrh-121048.

[27] Heman-Ackah YD, Dean CM, Sataloff RT. Strobovideolaryngoscopic findings in singing teachers. Journal of voice: official journal of the Voice Foundation. 2002;16(1):81–6. 10.1016/s0892-1997(02)00075-9. 10.1016/s0892-1997(02)00075-9 12002891

[28] Kwok M, Eslick GD. The impact of vocal and laryngeal pathologies among professional singers: a meta-analysis. J Voice. 2019;33:58–65. 10.1016/j.jvoice.2017.09.002. 10.1016/j.jvoice.2017.09.002 29523383

[29] Muniraju M, Chaithanya M. A study on videostroboscopic changes and voice analysis in professional voice users. Int J Otorhinolaryngol Head Neck Surg. 2025;11:126–30. 10.18203/issn.2454-5929.ijohns20250790.

[30] Brinca L, Nogueira P, Tavares AI, Batista AP, Gonçalves IC, Moreno ML. The prevalence of laryngeal pathologies in an academic population. J Voice. 2015;29(1):130.e1-130.e9. 10.1016/j.jvoice.2014.04.009. 10.1016/j.jvoice.2014.04.009 25008377

[31] Preciado-López J, Pérez-Fernández C, Calzada-Uriondo M, Preciado-Ruiz P. Epidemiological study of voice disorders among teaching professionals of La Rioja. J Voice. 2008;22(4):489–508. 10.1016/j.jvoice.2006.11.008. 10.1016/j.jvoice.2006.11.008 17574808

[32] Alshuhayb BS, Alkhars AZ, AlMaghlouth MK, Alkhars FS, Alamer ZA, Alarfaj AA, et al. Voice disorders among teachers in Al-Ahsa, Eastern Region, KSA: vocal complaints, treatment-seeking behaviors, and knowledge of vocal care. J Voice. 2025;39(1):147–57. 10.1016/j.jvoice.2022.07.001. 10.1016/j.jvoice.2022.07.001 35970654

[33] Lenell C, Sandage MJ, Johnson AM. A tutorial of the effects of sex hormones on laryngeal senescence and neuromuscular response to exercise. J Speech Lang Hear Res. 2019;62(3):602–10. 10.1044/2018_JSLHR-S-18-0179. 10.1044/2018_JSLHR-S-18-0179 PMC6802894 30950744

[34] Alves M, Krüger E, Pillay B, van Lierde K, van der Linde J. The effect of hydration on voice quality in adults: a systematic review. J Voice. 2019;33(1):125.e13-125.e28. 10.1016/j.jvoice.2017.10.001. 10.1016/j.jvoice.2017.10.001 29122414

[35] Bovo R, Trevisi P, Emanuelli E, Martini A. Voice amplification for primary school teachers with voice disorders: a randomized clinical trial. Int J Occup Med Environ Health. 2013;26(3):363–72. 10.2478/s13382-013-0115-1. 10.2478/s13382-013-0115-1 23817868

[36] Zabret M, Hočevar Boltežar I, Šereg Bahar M. The Importance of the Occupational Vocal Load for The Occurence and Treatment of Organic Voice Disorders. Slov J Public Health. 2018;57(1):17–24. 10.2478/sjph-2018-0003. 10.2478/sjph-2018-0003 PMC5894365 29651311

