# Supplementary material for: Understanding voice disorders among university staff: prevalence and associated factors
Source: BMC Public Health. 2026 Apr 13;26:1243. doi: 10.1186/s12889-026-27088-8 (PMC13085562; doi:10.1186/s12889-026-27088-8)
Supplement: Supplementary file 1 — Supplementary Material 1 [file 12889_2026_27088_MOESM1_ESM.pdf]

The questionnaire

This questionnaire assesses the prevalence of voice disorders among university staff members and the factors associated with them. Completion of this survey implies informed consent to participate in the study. All responses will be kept strictly confidential and used exclusively for scientific research purposes.

Note: **If you have experienced any of the following conditions, please do not complete the questionnaire:**

• Neck injury or accident
• Neck surgery, such as thyroid surgery, resulting in a change in voice
• Surgery that required general anesthesia and resulted in a change in voice
• Tumors in the laryngeal area

- Age (year):

- Gender: ◯ Male ◯ Female

- Marital status: ◯ Single ◯ Married ◯ Widow ◯ Divorced

- If you have children, please mention the number of children you have ………

- Residence: ◯ Urban ◯ Rural

- The name of your faculty:

- If you work at the Faculty of Medicine,

Your teaching subject is: ◯ Basic ◯ Clinical

- Your academic position: ◯ Teaching staff ◯ Assistant staff

- Years of employment: ……………………………………………………………….

- Number of teaching sessions per week: ……………………………………….

- Are you participating in remote learning? ◯ Yes ◯ No

- Do you use speakers (e.g. microphone)? ◯ Yes ◯ No

- Do you use a loud voice? ◯ Yes ◯ No

- Are you a smoker? ◯ Yes ◯ No

- Do you drink excessive tea (more than three cups per day)? ◯ Yes ◯ No

- Do you drink excessive coffee (more than three cups per day)? ◯ Yes ◯ No

- The number of cups of water drinking: …………………………………….

- Do you have Gastroesophageal Reflux Disease (GERD)? ◯ Yes ◯ No

- Do you have allergic diseases, especially respiratory system? ◯ Yes ◯ No

- Do you have chronic sinusitis or rhinitis? ◯ Yes ◯ No

- Do you have chronic cough? ◯ Yes ◯ No

- Do you have repeated respiratory tract infections? ◯ Yes ◯ No

- **Do you have a family history of voice disorders?** ◯ Yes ◯ No

- **Do you have a voice complaint?** ◯ Yes ◯ No

- If you have a voice complaint, it is

- **Hoarseness of voice** ◯ Yes ◯ No
- **Throat dryness** ◯ Yes ◯ No
- **Sore throat** ◯ Yes ◯ No
- **Frequent cleaning of voice** ◯ Yes ◯ No
- **Difficult breathing** ◯ Yes ◯ No
- **Sense of mass** ◯ Yes ◯ No
- **Difficult speaking** ◯ Yes ◯ No
- **Difficult swallowing** ◯ Yes ◯ No
- **Sudden suffocation** ◯ Yes ◯ No

- The consequences of this voice complaint on your professional life:

◯ No effect

◯ I had thought to leave work

◯ I take an absenteeism from work

- Did you do any of these methods to relieve your voice complaint?

◯ Excess intake of fluids

◯ Avoid shouting

◯ Avoid noisy environment

◯ Use speakers

◯ Visit a doctor

- If you didn’t visit a doctor, what was the cause?

◯ I do not have enough time

◯ It is not serious

◯ I don't know how or where to seek medical help

- Did you hear about voice care? ◯ Yes ◯ No

- What was your source of the information about voice care?

◯ Internet

◯ Family & friends

◯ Doctors visited for voice disorders

◯ Social media

◯ Training courses

◯ Books

- Are you ready to attend courses about voice care if available? ◯ Yes ◯ No
